# Limitations in the recording of maternal mortality in Germany: An analysis of statistical challenges

**DOI:** 10.25646/13382

**Published:** 2025-09-03

**Authors:** Safiya Fatima Zaloum, Julia Callaghan, Amira Goepfrich, Joachim Dudenhausen, Lars Paulson, Lars Hellmeyer, Klaus Vetter, Martina Ziegert, Thorsten Braun, Josefine Theresia Koenigbauer

**Affiliations:** 1 Department of Obstetrics, Charité University Hospital, Berlin, Germany; 2 Central Archive for Death Certificates, Berlin, Germany; 3 Registry of Mortality in Obstetrics, Berlin, Germany; 4 Department of Gynecology and Obstetrics, Vivantes Klinikum im Friedrichshain, Berlin, Germany; 5 Division of Experimental Obstetrics

**Keywords:** Maternal mortality, Pregnancy, Death certificates, Postpartum period, Berlin, Germany, Underreporting of maternal deaths, Public health, Maternal mortality registry, MMR, Quality assurance in obstetrics, Healthcare quality

## Abstract

**Background:**

The World Health Organization (WHO) defines maternal mortality as the death of a woman during pregnancy or up to 42 days after delivery. The maternal mortality ratio (MMR) serves as an indicator of the quality of health care. In Germany, recording is based on the death certificate (ICD-10 code), with variations in documentation leading to underreporting. Studies indicate insufficient data in Berlin and queries in Germany.

**Method:**

2,316 death certificates of women (aged 15 – 50) from the Berlin Central Archive (2019 – 2022) were analysed to identify maternal deaths and the quality of the information provided was assessed. In addition, the recording of pregnancy status on death certificates was examined nationwide.

**Results:**

Fourteen maternal deaths (excluding late cases according to the WHO) were identified. Only four cases were identifiable as maternal deaths solely on the basis of ICD-10 codes. The additional information ‘Is or was the woman pregnant?’ which is important for identification, was available in about a quarter of the death certificates reviewed. In 73.2 % of cases, the question ‘Is or was the woman pregnant?’ remained unanswered. A nationwide comparison of death certificates revealed considerable differences: only Bavaria and Bremen followed the WHO definition. Saxony-Anhalt does not record pregnancy status at all.

**Conclusion:**

The recording of maternal mortality in Germany is incomplete. Death certificates are often deficient. Many federal states record periods outside the WHO definition (3 – 12 months after birth). A standardized national system for registering maternal deaths is required to improve data collection and enable better prevention.

## 1. Introduction

Maternal mortality is considered a key indicator of the quality of a health system worldwide, as it reflects women’s access to adequate prenatal care, obstetric care, and medical services [1].

The World Health Organization (WHO) defines maternal mortality as the death of a woman during pregnancy, childbirth, or within 42 days of the end of pregnancy, regardless of the duration and location of the pregnancy, if the cause of death is related to or exacerbated by the pregnancy. Accidental or incidental deaths are excluded [2]. The maternal mortality ratio (MMR) is defined as the number of maternal deaths per 100,000 live births in the same period and is used for international comparison. It describes the risk of dying during pregnancy or shortly thereafter from pregnancy-related causes, with live birth serving as a proxy for pregnancy [3]. In addition, the WHO defines a late maternal death as the death of a woman from direct or indirect pregnancy-related causes between 43 days and one year after the end of pregnancy. The term ‘comprehensive maternal death’ (according to International Classification of Diseases: ICD-11) describes a collective category that includes both early and late maternal deaths [3, 4]. These classifications serve to improve the accuracy of recording and enable more targeted prevention of pregnancy-related deaths [3].

Maternal and late maternal deaths are further subdivided into direct and indirect deaths. Direct deaths result from obstetric complications (e.g., severe bleeding during childbirth), while indirect deaths are caused by pre-existing conditions or conditions exacerbated by pregnancy (e.g., severe pneumonia leading to death) [2].

The WHO estimates that in 2023, more than 700 women worldwide – about one woman every two minutes – will die in connection with pregnancy and childbirth [5]. Reducing maternal mortality was first established as a global goal in the Millennium Development Goals (MDGs) in 2000, with a target of reducing the MMR by 75 % by 2015 compared to 1990 [6]. Despite significant progress – the global MMR fell from 339/100,000 live births in 2000 to 227/100,000 in 2015 – this target was not achieved [7].

Building on the MDGs, the Sustainable Development Goals (SDGs) adopted in 2015 formulated a new target: to reduce the global MMR to < 70/100,000 live births by 2030 [8]. Since then, the global MMR has continued to decline from 223/100,000 in 2020 to 197/100,000 in 2023 – an improvement that nevertheless requires further progress to achieve the SDG target [5, 7].

The Organization for Economic Cooperation and Development (OECD) reported an MMR of 4.1/100,000 live births for Germany in 2022 [9]. This calculation is based on the recording of maternal deaths using the documentation of relevant ICD-10 diagnoses (O00-O99, excluding O96 and O97) on the death certificate and the number of live births from population statistics. The ICD diagnoses are taken from the chapter on pregnancy, childbirth, and the postpartum period. The diagnoses O96 and 97 code late maternal deaths [10].

After the physician issues a death certificate, it is forwarded to the registry office (non-confidential part) and then to the health department (confidential part). The doctor who performs the post-mortem examination is responsible for recording the immediate cause of death, the preceding cause, and the underlying condition. The health authorities forward relevant data to the state statistical offices, which use the ICD-10 coding system to determine the underlying condition, i.e., the underlying disease that led to death. The Federal Statistical Office (Destatis) carries out the central data evaluation as part of the cause of death statistics and reports the results at national level and to international organizations such as the WHO and OECD [11, 12]. The recording process in Germany is shown schematically in Figure 4.

**Key messages**

► Maternal mortality is a key indicator of the quality of medical care in a country.
► In Germany, maternal deaths are determined on the basis of ICD coding on death certificates. However, the documentation of essential ICD codes is often incomplete.
► The requirement on some death certificates to provide additional pregnancy-related information in women of childbearing age (e.g., on pregnancy, childbirth, termination, or miscarriage) is not uniformly regulated nationwide and has not yet been systematically taken into account in the recording of maternal deaths.
► Standardized, nationwide recording of maternal deaths is necessary to improve data quality and enable targeted measures to reduce maternal mortality.

**Figure 1:**
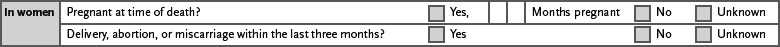
Berlin death certificate, additional question ‘In women’ regarding an existing or terminated pregnancy in the last three months. Source: Senate Department for Justice and Consumer Protection [13]

The Berlin death certificate also contains an additional entry ‘In women,’ which consists of two sub-questions and asks about an existing pregnancy or childbirth, a miscarriage (pregnancy loss), or an abortion in the last three months (Figure 1) [13]. However, this information is not included in the official maternal mortality statistics, which rely solely on the above-mentioned ICD codes. The most common causes of maternal mortality worldwide include: Severe bleeding (e.g., after childbirth or due to a ruptured ectopic pregnancy), high blood pressure during pregnancy (e.g., preeclampsia, eclampsia), infections, thrombosis, and embolisms, as well as complications from pre-existing conditions (e.g., cardiovascular disease) [5]. In many cases, these causes are preventable or treatable if detected in time [14]. It is therefore important that deaths related to pregnancy and childbirth are recorded completely, accurately, and in a differentiated manner. This approach enables the identification of systemic weaknesses within the healthcare system and supports the implementation of targeted improvements, for example in early detection, clinical decision-making, and emergency management.

Additional data is provided by the Institute for Quality Assurance and Transparency in Health Care (IQTIG). This institute is responsible for quality assurance in inpatient obstetric care and uses a quality assurance form (QS form) for this purpose, which records, among other things, the death of the mother during her hospital stay for childbirth [15]. A limitation of this recording is that it captures and analyses only those deaths occurring during hospitalisation in association with childbirth. For Germany, an MMR of 5.4/100,000 live births was documented in 2021, 4.2/100,000 in 2022, and 3.2/100,000 in 2023 in the context of inpatient obstetric care [16]. The difference could be explained by the different types of case recording (OECD up to 42 days after birth, IQTIG only in inpatient settings during childbirth).

Recent studies suggest that maternal mortality in Berlin and Germany may be underestimated. Callaghan et al. were able to show that in the period from 2019 to 2022, only four maternal deaths in Berlin were clearly identifiable using an appropriate ICD-10 code. However, the death certificates included 10 additional maternal deaths that were only identifiable as clear cases through the additional information ‘In women’ or confidential inquiries as well as file access [17]. Possible causes include incomplete queries of relevant data and insufficient information on death certificates.

The aim of this study is to analyse the quality of maternal mortality recording in Berlin based on the additional information ‘In women’ on death certificates. Particular attention is paid to the quality of documentation. The basis for this analysis is a data set of 2,316 death certificates from the preliminary work of Callaghan et al. [17].

In addition, state-specific differences in the design of the additional question ‘In women’ on the respective death certificates are examined.

The results can help raise awareness of the incomplete and inconsistent recording of these rare serious events and serve as a basis for optimising the recording of maternal deaths in Germany in the future.

## 2. Method

### 2.1 Data basis and study design

This study consists of two parts. The first part examines the recording of maternal deaths on Berlin death certificates, with a particular focus on the quality of the additional question ‘In women.’ The second part compares the wording of the additional question ‘In women’ in the individual federal states.

### 2.2 Quality of completion of the additional information ‘In women’

In the first part of the study, death certificates from the Central Archive for Death certificates (ZfL) in Berlin were evaluated. This archive records all deaths in Berlin, regardless of the place of residence of the deceased. The data used here has already been used in the work of Callaghan et al. [17], but there it was analysed and published exclusively with regard to maternal deaths. While the aforementioned study focused on the number of maternal deaths, the current analysis deals with the quality of the information in the additional field of the post-mortem examination certificates.

The data set includes deaths of women aged 15 to 50 in the period from January 1, 2019, to December 31, 2022 (n = 2,316). Potential maternal deaths were identified on the basis of the additional information ‘In women,’ relevant ICD-10 diagnoses (O00 – O99), and other medical information on the death certificates (epicrisis/free text). This was supplemented by confidential inquiries to the persons who completed the forms. The recorded cases (n = 23) were classified as ‘maternal death,’ ‘late maternal death,’ and ‘non-pregnancy-related death’ according to WHO criteria.

### 2.3 Additional information ‘In women’ on the death certificates of the federal states

In the second part of this study, a document-based cross-sectional analysis was conducted to evaluate death certificates as a method for recording maternal deaths. The focus was on the design of the additional information ‘In women’ and state-specific differences in the query (Figure 1). For this purpose, the death certificates of all 16 federal states were analysed.

The death certificates were obtained through targeted requests to health authorities and associations of statutory health insurance physicians in the federal states, as well as through research of publicly available online sources, in particular the state portals.

The criteria for the analysis included:

1. the presence of a specific additional note in women,
2. the query of an existing pregnancy at the time of death,
3. recording of a postpartum death,
4. the number of days after the end of a pregnancy.

The consideration of the postpartum period was particularly relevant, as this is an essential prerequisite for compliance with the WHO definition of maternal mortality (42 days after the end of pregnancy).

### 2.4 Statistical analyses

The statistical analyses were performed using IBM SPSS Statistics 30 and Microsoft Excel 365. The analysis was based on two data sets: one from Callaghan et al. on identified deaths associated with pregnancy in Berlin [17], and the other from the data collected on death certificates in the federal states.

The quality of the information provided on the post-mortem examination forms and the additional questions was analysed using descriptive statistics, including frequency distributions and cross-tabulations. Differences in the recording of maternal deaths between the federal states were also examined.

## 3. Results

### 3.1 Recording of maternal deaths using the additional question ‘In women’

**Figure 2:**
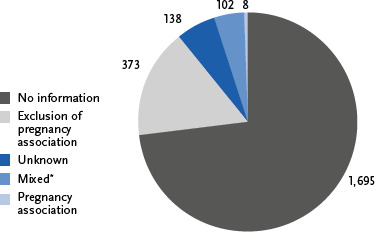
Responses to the additional question about women on death certificates in Berlin (n = 2,316, 2019 – 2022). The figures show the percentages and number of cases for each response category: no information (73.2 %), pregnancy ruled out (16.1 %), unknown (6.0 %), mixed^*^ (4.4 %), and pregnancy association (0.35 %). ^*^‘Mixed’ = different answers to the two sub-questions (e.g., ‘no’ and ‘unknown’); excluding cases with ‘yes.’

Of the 2,316 death certificates analysed, 1,695 cases (73.2 %) did not contain any information on the additional question and could therefore not be further evaluated. In the remaining 621 cases (26.8 %), at least one additional piece of information on an existing or terminated pregnancy was provided. The majority of these cases (n = 511) contained the answer ‘no’ twice (exclusion of a pregnancy association). In 102 cases, there were mixed combinations of ‘no,’ ‘unknown,’ or no information provided (mixed). Only in eight cases was the answer option ‘yes’ given at least once and could therefore be included in the further evaluation (pregnancy association) (Figure 2).

**Figure 3:**
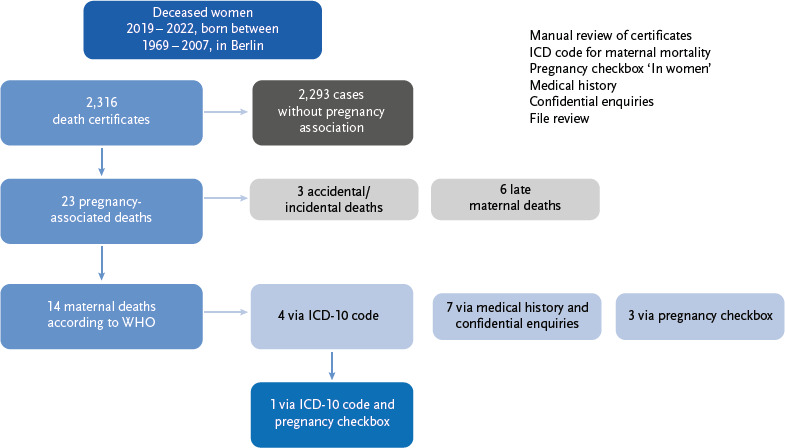
Overview of the systematic recording of maternal deaths (n =14) from a total of 2,316 death certificates issued by the Berlin Medical Examiner’s Office in women of childbearing age. The classification as pregnancy-related or maternal death was made by manual review, supplemented by ICD-10 coding, additional information ‘In women,’ evaluation of the epicrisis, file review, and confidential inquiries. ^*^WHO = World Health Organization; ICD = International Classification of Diseases

Through the comprehensive evaluation of 2,316 death certificates in Berlin between 2019 and 2022 – including ICD codes, additional information such as ‘In women,’ confidential inquiries to the issuers, and, in some cases, supplementary file access – a total of 23 pregnancy-related maternal deaths were identified (Figure 3).

These included three accidental deaths (e.g., due to accidents) and six late maternal deaths, i.e., deaths that occurred between 43 days and one year after birth.

Fourteen cases met the criteria for maternal death according to the WHO definition. In many cases, they could only be identified through additional data sources. The analysis showed that only a quarter of these cases (4 out of 14) had a relevant ICD diagnosis and were therefore included in national and international statistics.

The remaining ten cases were recorded using supplementary information on the death certificates:

► five cases via free text in the epicrisis,
► three cases via the additional information ‘In women,’
► two cases through confidential inquiries to the issuers.

One case could be clearly assigned both through correct ICD coding and additional information (Figure 3).

Based on the 23 deaths associated with pregnancy, eight of these deaths could be identified using the additional information ‘In women’ on the death certificate. However, it was found that not all eight cases were classified as maternal deaths. Only four of the eight cases with the additional information checked met the WHO criteria for maternal mortality. The remaining four were classified as late maternal deaths (n =1), accidental/unintentional deaths (n = 2), and unclassified cases (n = 1). This distribution illustrates that not every additional information necessarily indicates a maternal death according to the WHO definition and that recording additional information alone could potentially lead to overrecording.

### 3.2 Analysis of the additional question ‘In women’ on the death certificates of the federal states

With regard to the additional information ‘In women,’ the analysis showed variability between the death certificates of the German federal states in terms of the recording and documentation of potential maternal deaths. The investigation was based on the WHO criteria, which define the relevant period from pregnancy to 42 days postpartum. For all 16 federal states, the respective death certificates were identified through targeted research and included in the analysis. However, a complete survey of all formats used in Germany is not possible due to the lack of a central recording office and inconsistent publication practices.

The results show differences between the federal states in the periods recorded after the end of a pregnancy. Bavaria and Bremen are the only federal states that record a period of exactly 42 days, thus complying with the criteria defined by the WHO.

In eleven of the 16 federal states, including Berlin, a period of three months is specified, while Hesse and North Rhine-Westphalia record an extended period of 365 days or one year. Saxony-Anhalt is an exception, as it is the only federal state that does not ask additional questions ‘In women’ on the death certificate (Table 1). In all other 15 federal states, questions are asked about both possible pregnancy at the time of death and postpartum death.

Bavaria is the only federal state that records both maternal and late maternal mortality according to the WHO definition with an additional question that explicitly covers the period from 43 to 365 days after the termination of pregnancy.

Analysis of the additional questions reveals inconsistencies in both the time period covered and the wording, and consequently also in the recording of the reasons for termination of pregnancy. Twelve of the 16 federal states specifically record a delivery, abortion, or termination. Bavaria is also the only federal state that additionally asks about extra-uterine pregnancy (pregnancy in which the fertilized egg implants outside the uterine cavity). Saxony asks a more general question about whether there were signs of pregnancy in the last three months, while North Rhine-Westphalia extends this period to the last twelve months. As already mentioned, Saxony-Anhalt does not ask such additional questions.

## 4. Discussion

**Figure 4:**
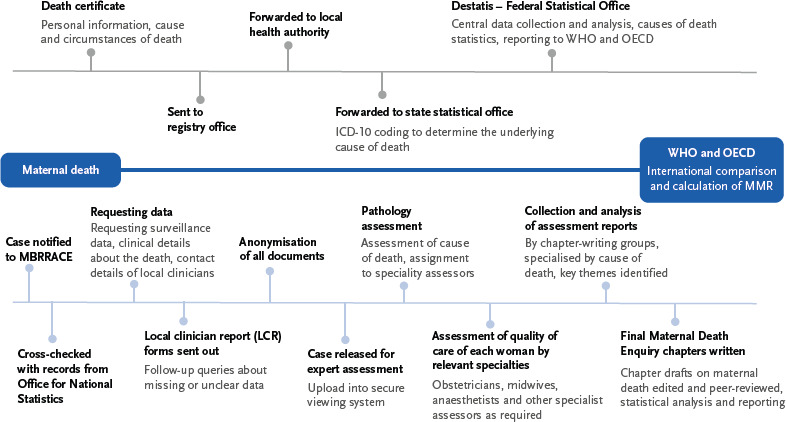
Schematic comparison of the recording steps for maternal mortality cases in Germany (top line) [9, 10] and UK-MBRRACE (bottom line) [26, 27]. The starting point ‘maternal death’ and the goal of reporting to ‘WHO and OECD’ are common to both. ^*^WHO = World Health Organization; OECD = Organisation for Economic Co-operation and Development; ICD-10 = International Statistical Classification of Diseases and Related Health Problems, 10th Revision; MMR = maternal mortality ratio; UK-MBRRACE = Mothers and Babies: Reducing Risk through Audits and Confidential Enquiries across the UK

There are significant gaps in the recording of maternal deaths in Germany. Maternal deaths are primarily identified in two ways: through quality assurance for hospital births and through the death certificate. In hospitals, documentation in the quality assurance form is mandatory, while recording via the death certificate is only possible if a corresponding ICD diagnosis is coded (Figure 4).

However, identifying maternal deaths solely on the basis of ICD diagnoses has proven to be insufficient. The quality of documentation on death certificates is inconsistent, and the design of the forms varies considerably between federal states. In particular, the additional question ‘In women’ varies greatly and often does not meet WHO criteria.

Of 2,316 death certificates analysed in Berlin (2019 – 2022), 26.8 % contained additional information on pregnancy. A total of 23 pregnancy-related deaths were identified, 14 of which were classified as maternal deaths according to the WHO definition. Only four of these cases were statistically identifiable based on ICD coding; the rest were identified via free text entries, additional fields (‘In women’) or follow-up questions. The analysis shows that additional information is helpful but not reliable: only four out of eight cases with the corresponding marking met the WHO criteria.

A nationwide comparison of death certificates also revealed considerable differences in the recording of pregnancy-related deaths: only Bavaria and Bremen adhere strictly to the WHO definition (42 days postpartum), while other federal states deviate significantly in terms of the recording period or the questions asked. Saxony-Anhalt does not collect any additional information on pregnancy. This heterogeneity makes it difficult to obtain valid and comparable nationwide data on maternal mortality.

An analysis of Berlin death certificates from 2019 to 2022 reveals a significant underreporting of maternal deaths. Only one-third of the 14 maternal deaths identified were recorded with relevant ICD diagnoses and included in the official statistics. These results are consistent with earlier studies, such as the investigation by Hellmeyer et al. [14], which initially recorded two maternal deaths for 2016 but identified three additional cases through further research.

The study by Welsch et al. in Bavaria [18] reveals a similar problem. By linking death certificate data, the Bavarian Perinatal Database, and confidential reports, it was possible to achieve a more precise classification of maternal deaths and to identify and analyse temporal trends. These findings provide valuable insights for improving obstetric care and underscore the need for expanded data collection.

In addition, general studies on the quality of death certificates reveal considerable shortcomings in medical documentation. An analysis from Mecklenburg-Vorpommern revealed a high error rate in both content and form [19]. Although all licensed physicians are authorised to issue death certificates, these results indicate a need for action to ensure the quality of post-mortem examinations, for example through mandatory continuing education [20]. In addition to the issuing medical profession, health authorities also have a central role to play: as the only systematic control authority to date, they can check death certificates for formal completeness and plausibility of content, thereby contributing to the correction of incorrect information and the clarification of cause of death statistics [21]. The additional lack of standardisation and digitisation exacerbates the structural deficits and contrasts with the high information potential of a correctly documented post-mortem examination: it forms the basis for comprehensive statistics on causes of death and is essential for epidemiological research and health policy decisions [20].

The results show that there is a need for action with regard to medical post-mortem examinations and argue in favour of structured, nationwide uniform recording of pregnancy-related information – regardless of the final coding by the state statistical offices.

The analysis of death certificates shows that the lack of a standardised federal form leads to significant differences in the additional information ‘In women’ between the federal states. While only Bavaria and Bremen record postpartum deaths according to WHO criteria with an explicit time period of 42 days, more than three-quarters of the federal states specify a longer period in their surveys, and Saxony-Anhalt does not ask for this additional information at all.

Since only ICD diagnoses are currently included in official statistics, the potential of the additional information remains untapped. However, this study shows that three of the 14 maternal deaths identified were recorded exclusively through the additional information. Only one case contained both an ICD- code and the additional information ‘in women,’ which made it possible to clearly classify it as a maternal death. This underscores the importance of the additional information as a supplementary recording method.

However, nationwide standardisation is necessary for the effective use of this additional question in the future. The current heterogeneity makes systematic evaluation difficult and significantly impairs the recording of maternal mortality.

### International approaches to improving the recording of maternal deaths

International experience shows that a standardised additional information can help identify maternal deaths. In the US and Taiwan, the implementation of a pregnancy checkbox led to an increase in the recorded maternal mortality rate. However, experience in the US and Taiwan also shows that the use of the additional question can lead to possible overrecording [22, 23]. These results are also found in the present project. For reliable data collection, it is therefore essential to combine different data sources. In their work, Welsch et al. have proposed a system that links death certificate data with perinatal and hospital data [18, 24].

Another promising approach for expanded recording is the Enhanced Obstetric Surveillance System (EOSS), which has already been implemented in several countries [24]. These systems link birth and death registers, hospital data, and data from death certificates to assess in detail the causal relationship between pregnancy and cause of death. Confidential and anonymised review of cases by multidisciplinary audit committees not only improves data quality but also leads to targeted recommendations for optimising obstetric care [25].

The standardised system for recording maternal deaths in the United Kingdom, which has been in place since 1952, is considered exemplary for the recording of maternal deaths. Under the UK MBRRACE system (Mothers and Babies: Reducing Risk through Audits and Confidential Enquiries across the UK), maternal deaths are anonymised and systematically investigated (‘Confidential Enquiries’). This uses a complex procedure of confidential inquiries to systematically and confidentially analyse maternal deaths and regularly identify specific gaps in care [26]. The latest report from 2020 – 2022 showed that in many women, significant symptoms were not investigated further but were incorrectly attributed to pregnancy. As a result, many deaths from venous thromboembolism occurred in early pregnancy, often due to a lack of early diagnosis and failure to initiate thrombosis prophylaxis. In the case of malignant diseases (cancer), inadequate diagnosis and delayed treatment were also repeatedly observed, as pregnancy was mistakenly considered an exclusion criterion. These examples illustrate how systematic case analyses are used in the United Kingdom to further develop evidence-based standards of care. National reports with specific recommendations for action are published every three years [27].

In comparison, Germany lacks a centralised and standardised system for recording, analysing and evaluating maternal deaths. Figure 4 shows the differences between the German recording system and the UK MBRRACE approach.

This study has some limitations that must be taken into account when interpreting the results. The analysis of death certificates is based exclusively on data from Berlin, which means that the results cannot be readily transferred to the whole of Germany. Comparative studies in other federal states would be necessary to test the generalisability. Due to the limited data available (including incomplete recording and a lack of valid case numbers for certain periods), it was not possible to calculate a reliable statistical maternal mortality rate (MMR). The aim of the analysis was therefore not to determine an exact MMR, but to identify trends and possible systematic underestimations prior to retrospective case review.

Another methodological problem lies in the overall limited data available on maternal mortality in Germany. Apart from the results of this study, there is only the study by Welsch et al. [18] for Bavaria and two further studies on maternal mortality in Berlin [14, 17]. The small number of comparable studies makes it difficult to comprehensively classify the results.

In addition, the inconsistent design of death certificates posed a challenge – both nationwide and within individual federal states. For example, the health department of the Harz district reported that several versions of death certificates were in use in Saxony-Anhalt. Within the scope of this study, it was not possible to obtain a complete overview of the different formats used in all federal states. It therefore remains unclear whether this variability within a federal state is an isolated case or a more widespread problem.

A key problem in identifying maternal deaths via death certificates is the lack of systematic recording of the pregnancy context, even when relevant data fields such as the additional question ‘In women’ are provided. The present analysis shows that this additional information was not filled in in over 70 % of cases, even though it can be essential for identifying maternal deaths. Even if corresponding fields are introduced nationwide, it is therefore to be expected that a significant number of maternal deaths will remain undetected if no structured collection, control, and follow-up mechanisms are established. This highlights the limitations of a purely form-based survey. Valid identification would only be possible through systematic evaluation of the additional information in conjunction with further information, such as free text fields, clinical context data, or an audit system such as EOSS. Without these steps, underreporting will remain structural.

For Germany, an obligation to complete the additional question ‘In women’ should be considered. This would allow this question to be used in all federal states to determine maternal deaths. Regular training on how to complete death certificates correctly can significantly improve data quality and contribute to more accurate recording of maternal deaths.

## Figures and Tables

**Table 1:** Time periods specified by the federal states for recording postpartum deaths (n =16 federal states) in the additional question on the death certificate, including WHO compliance (≤ 42 days), query on later maternal mortality, query on the reasons for termination of pregnancy.

Federal state	Period in days	WHO criteria fulfilled	Late maternal death	Delivery inquiry	Miscarriage inquiry	Abortion inquiry	Ectopic pregnancy inquiry
Baden-Wuerttemberg	90						
Bavaria	42						
Berlin	90						
Brandenburg	90						
Bremen	42						
Hamburg	90						
Hesse	365						
Mecklenburg-Vorpommern	90						
Lower Saxony	90						
North Rhine-Westphalia	365						
Rhineland-Palatinate	90						
Saarland	90						
Saxony	90						
Saxony-Anhalt	-						
Schleswig-Holstein	90						
Thuringia	90						
